# Preventing mood and anxiety disorders in youth: a multi-centre RCT in the high risk offspring of depressed and anxious patients

**DOI:** 10.1186/1471-244X-12-31

**Published:** 2012-04-17

**Authors:** Maaike H Nauta, Helma Festen, Catrien G Reichart, Willem A Nolen, A Dennis Stant, Claudi LH Bockting, Nic JA van der Wee, Aartjan Beekman, Theo AH Doreleijers, Catharina A Hartman, Peter J de Jong, Sybolt O de Vries

**Affiliations:** 1 Department of Clinical Psychology, University of Groningen, Grote Kruisstraat 2/1, 9712 TS, Groningen, The Netherlands; 2 Curium/Leiden University Medical Center, Endegeesterstraatweg 27, 2342 AK, Oegstgeest, The Netherlands; 3 Department of Psychiatry/Interdisciplinary Center of Pathology of Emotion, University Medical Center Groningen, University of Groningen, Hanzeplein 1, 9700 RB, Groningen, The Netherlands; 4 Department of Epidemiology, University Medical Center Groningen, University of Groningen, Hanzeplein 1, 9700 RB, Groningen, The Netherlands; 5 Department of Psychiatry and Leiden Institute for Brain and Cognition, Leiden University Medical Center, Albinusfreef 2, 2333 ZA, Leiden, The Netherlands; 6 Department of Psychiatry and EMGO institute, VU University Medical Center/GGZ inGeest, A.J. Ernststraat 1187, 1081 HL, Amsterdam, The Netherlands; 7 de Bascule/Academic Medical Center Amsterdam, p/a Postbus 303, 1115 ZG, Duivendrecht, the Netherlands; 8 Mental Health Care Friesland (GGz Friesland), Borniastraat 34B, 8934 AD, Leeuwarden, The Netherlands

**Keywords:** Prevention, Offspring, Anxiety, Depression, Randomised controlled trial, Cost effectiveness

## Abstract

**Background:**

Anxiety and mood disorders are highly prevalent and pose a huge burden on patients. Their offspring is at increased risk of developing these disorders as well, indicating a clear need for prevention of psychopathology in this group. Given high comorbidity and non-specificity of intergenerational transmission of disorders, prevention programs should target both anxiety and depression. Further, while the indication for preventive interventions is often elevated symptoms, offspring with other high risk profiles may also benefit from resilience-based prevention programs.

**Method/design:**

The current STERK-study (Screening and Training: Enhancing Resilience in Kids) is a randomized controlled clinical trial combining selected and indicated prevention: it is targeted at both high risk individuals without symptoms and at those with subsyndromal symptoms. Individuals without symptoms meet two of three criteria of the High Risk Index (HRI; female gender, both parents affected, history of a parental suicide (attempt). This index was developed in an earlier study and corresponds with elevated risk in offspring of depressed patients. Children aged 8–17 years (n = 204) with subthreshold symptoms or meeting the criteria on the HRI are randomised to one of two treatment conditions, namely (a) 10 weekly individual child CBT sessions and 2 parent sessions or (b) minimal information. Assessments are held at pre-test, post-test and at 12 and 24 months follow-up. Primary outcome is the time to onset of a mood or anxiety disorder in the offspring. Secondary outcome measures include number of days with depression or anxiety, child and parent symptom levels, quality of life, and cost-effectiveness. Based on models of aetiology of mood and anxiety disorders as well as mechanisms of change during interventions, we selected potential mediators and moderators of treatment outcome, namely coping, parent–child interaction, self-associations, optimism/pessimism, temperament, and emotion processing.

**Discussion:**

The current intervention trial aims to significantly reduce the risk of intergenerational transmission of mood and anxiety disorders with a short and well targeted intervention that is directed at strengthening the resilience in potentially vulnerable children. We plan to evaluate the effectiveness and cost-effectiveness of such an intervention and to identify mechanisms of change.

**Trial registration:**

NTR2888

## Background

Anxiety disorders are highly prevalent among children and adolescents with estimates of 11.6% year prevalence in adolescents alone [1], and depression is highly prevalent among adolescents, with estimates of 3.8% year prevalence [1]. Anxiety and mood disorders in childhood and adolescence not only have a high impact on present functioning [2,3], but are also associated with long-term negative consequences [4,5]. In the Netherlands alone, estimations are that as many as 37.400 adolescents (3.8%) suffer from depressive disorder [1], corresponding with a burden of disease of 7900 Disability Adjusted Life Years (DALYs), meaning that per year 7900 healthy years are lost due to depression alone in youngsters ‘Gezond Verstand’, [6]. For anxiety disorders, estimates are 113.000 adolescents (11.6% year prevalence) and 15.000 DALYs.

From epidemiological research, we know that anxiety and mood disorders often run in families: the incidence of depression and anxiety is elevated by a factor 2–6 among offspring of patients with such a disorder (e.g. [7,8]). There is considerable aetiological and phenomenological overlap between mood and anxiety disorders. Anxiety often precedes depression [9] with the age of onset of depression being typically 5 to 10 years later than that of anxiety disorders e.g. [10].

Given the high prevalence of anxiety and mood disorders, the high impact on individuals as well as the associated societal costs, there is a clear need for prevention of anxiety and mood disorders in youth. Since these disorders run in families, the family may be a good starting point for prevention.

During the last two decades, a variety of programs has been developed to prevent anxiety disorders or depression among children and adolescents [overview: [2,11]. The results of universal prevention programs are disappointing for both anxiety and mood symptomatology [e.g. [2,12]. For selective prevention (targeting high risk groups) and indicated prevention (targeting those with subclinical symptoms) results are more promising.

Despite of the relatively high risk in offspring, thus far the number of randomised controlled trials testing the efficacy of indicated prevention is very limited: Four randomised trials have aimed at offspring of depressed patients [13-16], with two studies reporting on cognitive behavioural group treatments for offspring, and two studies including more family-based treatments. Only one study reported on prevention in children of anxiety disordered parents [17].

In the first study, the effectiveness of the 15-session cognitive group training ‘Coping with stress’ was examined in adolescent offspring of a depressed parent [13]. Adolescents (N = 94) were aged 13–17 years and had subclinical depressive symptoms or a history of depression themselves. The program encompassed cognitive restructuring techniques aimed at changing maladaptive thoughts in general and dysfunctional thoughts with regard to having a depressed parent. The intervention was not only effective in reducing depressive symptomatology, but also showed a significant reduction of new depressive episodes relative to care as usual. The program was also cost-effective [18].

Second, Garber and colleagues designed the largest multicenter trial in this field, including 316 youngsters aged 13–17 years. They were offspring of a parent with a current or prior depressive disorder, and had had a depression themselves or reported depressive symptoms. Adolescents were randomized to either care as usual or an 8-session CBT group training existing of cognitive restructuring and problem solving. Parents were invited to two parent information sessions. Adolescents were less likely to suffer from a depressive episode if they had received the training (21% versus 32% onset in 6 months), but only if the parent was remitted at the time of the intervention.

The third study investigated the effectiveness of a family program, including the 8–15 year-old offspring of at least one parent with an episode of depression in the past 18 months [16]. At least one of the children in the family needed to be free of a depressive disorder. The 6–11 session family program was compared to two plenary group lectures for parents. Both interventions advocated open discussion about the parental illness and were directed at change in family dynamics. Both interventions proved equally effective in increasing family functioning and decreasing internalising behaviours up to 4.5 year follow-up (based on 105 families) [18]. Families in the more intensive treatment reported more benefits in parent-child behaviours and regarding the child’s understanding of parental mental illness.

The fourth trial also investigated the effectiveness of a family intervention for families with at least one parent with a history of depression [19]. The children did not need to report symptoms themselves to participate in the study. Participants included 111 families with 155 children aged 9–15 years, who were randomized to either written information only or to a 12-session cognitive behavioral family intervention in a group format [19]. The family intervention focused on enhancing awareness of the role of depression in a family, on ameliorating parent–child interactions by teaching parenting skills (focusing on parental warmth and structure), and on learning general coping skills (for parents and children separately). Results indicate that children in the family program showed more benefits in terms of internalizing and externalizing symptoms in both parent and child reports. These gains were maintained at two year follow-up [20].

The only trial so far focusing on offspring of patients with anxiety disorders included 40 children aged 7–12 years, who were randomized to either an 8-session family-based CBT program focusing on coping and strengths or to a waitlist control condition. Children were not allowed to have a current anxiety disorder. Current or past symptomatology was not warranted for inclusion in the study. This trial was successful in preventing the onset of anxiety disorders in the offspring: offspring in the active condition did not develop anxiety disorders, whereas 30% of waitlist children met criteria for an anxiety disorder at 1 year follow-up.

In conclusion, results on interventions for offspring of depressed patients (4 studies) and anxious patients (1 study) were positive overall, including benefits on offspring symptomatology [20,21], offspring onset of disorder [13,14,17,20], parent‐child interaction [21], and offspring knowledge on the parent’s illness [21].

The current study builds upon these studies, while adding to them in a variety of ways: (1) we use additional risk factors to select ultra high risk individuals among offspring of patients with a mood or anxiety disorder; (2) we focus on both depression and anxiety; (3) we aim at symptom reduction as well as at increasing strengths and resilience; (4) we include mediators and moderators of change; (5) we include short and long-term cost-effectiveness analyses.

The aforementioned studies were either indicated prevention programs (youth with elevated symptoms) or selective prevention programs (youth with a high risk because of parental illness only). In our study, we aim at combining the two and thus selecting ultra high risk offspring. In line with earlier studies, we select youth with current symptomatology (of anxiety or mood). In addition, we wanted to make a selection of the symptom-free children. We know that some of the offspring may develop disorders over time, even though they currently do not report such symptoms. Recently, we have developed a prognostic index that predicts the development of anxiety or mood disorders in offspring (High Risk Index (HRI; de Vries, Landman-Peeters, Burger, Reichart, Nauta, den Boer, Nolen, Ormel, & Hartman: Predicting mood- and anxiety disorders in offspring of patients with a depressive disorder, unpublished manuscript)). This was done on the basis of a study examining offspring (n = 434) of patients with a unipolar mood disorder in a large prospective study, the ARIADNE-cohort (Adolescents at Risk of Anxiety and Depression, and Neurobiological and Epidemiological approach [22]). Three factors were associated with an increased risk of developing anxiety or mood disorders: female sex, having two affected parents, and suicide attempt(s) of one of the parents. In children with two or three risk factors (20% of the sample) the cumulative incidence of mood and anxiety disorders was 70% at the age of 20. In children with one or no risk factor, percentages dropped to 45% and 25% respectively. In the current study, inclusion is therefore based on the HRI as well as on symptomatology. Thereby, this study uniquely combines selected and indicated prevention [23].

For this group of high-risk offspring, we developed an individual cognitive behavioural therapy (CBT) based intervention targeting multiple risk and protective factors known to be associated with the onset of anxiety and depression (as recommended by Cuijpers [24]). Many prevention programs have focused on symptom reduction, whereas training of positive aspects and building resilience may be of utmost importance in prevention. Some at risk children may not show any symptomatology yet, and they may especially benefit from interventions focusing on strengths rather than vulnerabilities. Important protective factors in offspring include having knowledge on parental illness [25], having a supportive social network [22,26] including a non-ill parent if available [27], as well as displaying active coping skills and flexibility in coping style across situations [28]. Therefore, the offspring intervention includes psychoeducation for offspring, psychoeducation for parents, a focus on the social network, and problem solving skills training. With regard to the risk factors, we know that offspring may have a cognitive vulnerability in information processing: they report more negative and less positive self-statements [29]. Interventions focusing on positive self-statements, positive emotions and positive events may enhance resiliency and may function as a buffer against developing negative mood or anxious feelings. A final risk factor are subclinical complaints. These symptoms are addressed by regular behavioural interventions, namely exposure exercises for anxiety and behavioural activation for depressive symptoms. Behavioural activation may be of particular importance, since engaging in activities and relationships outside of the home environment has been found to be an important protective factor in adolescent offspring [25].

So far, most prevention interventions were group-based. Individual programs allow for more tailoring to the specific needs, strengths and weaknesses of the child. This is crucial because of the heterogeneity of the target population (children with anxiety, or depressive symptoms, or no symptoms but an elevated score on the High Risk Index). In addition, living with a depressed suicidal parent differs in many ways from living with anxious dependent one, for example. An individual approach allows the therapist to tailor the intervention also to the specific background of the child.

To date, preventive interventions have been designed to prevent either mood disorders or anxiety disorders. Keeping in mind the significant overlap in symptoms between depression and anxiety disorders, prevention studies should focus on both. Indeed, epidemiological studies have found that elevated symptoms of anxiety and depression occur in offspring of both anxious [30] and depressed patients [31]. Since the aetiology and pathogenesis of anxiety and depression also have considerable overlap, including offspring from patients with both mood and anxiety disorders provides the opportunity to study common mediating factors.

From a societal point of view, it is important to study the economic impact of psychiatric illnesses and possible effects of prevention programs, to assist future policy making and resource allocation. The economic evaluation in our study will focus on the differential effects on short and long term costs and health outcomes of the treatment conditions under study: CBT or minimal information. The study will be conducted from a societal perspective [32].

Treatment outcome studies typically include variables to study the treatment mechanisms (“how does the treatment work”) as well as the moderators of treatment outcome (“for what groups does the treatment work”)[33]. With regard to mediating factors in the current study, the intervention will aim at changing coping behaviour [34], increasing activities [35], and enhancing trust in the availability of attachment figures (in the social network). Even though we do not address cognitions directly through cognitive restructuring, we nonetheless want to investigate if the child’s attributional style changed through our behaviour intervention.

Moderating factors associated with (non)response are current parental psychopathology [14] and child’s symptomatology (internalising and externalising). We additionally measured some stable child characteristics that have been associated with the onset of anxiety or mood disorders, but have not been studied in relation to treatment outcome in offspring, namely reactive and regulative temperament [36], general executive functioning, and automatic self-associations [37]. In addition, some of the presumed mediating factors may also function as moderators.

In summary, even though there is extensive evidence for the intergenerational transmission of anxiety and mood disorders, few preventive intervention studies in offspring have been carried out. Our study adds to the current state of the art in combining selective and indicative prevention, to focus on both anxiety and mood disorders in adult patients, to focus on both anxiety and depression symptoms in offspring, to work on both symptom reduction and resilience, to study cost-effectiveness, and to examine mediators and moderators of outcome.

### Trial objective and purpose

The primary goal is to investigate whether a brief (10 + 2 sessions) cognitive behavioural treatment program on resilience and symptom reduction can prevent the incidence of depression or anxiety disorder in an ultra high risk sample of 8–18 year old offspring of patients with unipolar depression or anxiety disorder (sample defined by a High Risk Index or subclinical symptoms, or both). The second goal is to examine whether this intervention meets current standards for cost-effectiveness. A third goal is to explore the role of a number of factors that may potentially mediate or moderate the effect of the intervention. Mediating factors include coping, attributional style, daily activities, and optimism, while the selected moderating variables are child temperament characteristics and executive functioning, parental psychopathology (of both parents), child symptomatology, attachment, and (automatic) self-associations.

## Methods/design

The present project is designed as a selected and indicated prevention program: children and adolescents at high risk for developing affective disorders will be identified and treated if they run an ultra high risk. Such an approach results in a relatively powerful design [24]. The study is further designed as a randomised controlled trial (RCT), including an intervention condition and a minimal information condition.

### Participants

Participants in the study are children of patients with and anxiety or mood disorder. We aim at screening 554 children (T0), and at including 204 children in the intervention phase of the study. We have designed in- and exclusion criteria for parents and children:

Inclusion criteria (parents):

At least one of the biological parents, currently or in the past five years, treated for a unipolar mood disorder or an anxiety disorder; informed consent.

Exclusion criteria (both parents):

Mental retardation; severe alcohol or substance use disorder; schizophrenia or other primary psychotic disorder; schizoaffective disorder; bipolar disorder.

Inclusion criteria for the intervention phase (offspring): age 8–17; being at ultra high risk for developing a mood or anxiety disorder. Two pathways lead to the qualification of “ultra high risk”: (1) children report elevated symptoms of anxiety or depression, as defined as the 80^th^ percentile of either the subscale Depression or the cluster of subscales of Anxiety on the RCADS self-report. We used data from the large Dutch epidemiological study TRAILS to set these cut-offs at the various age segments (Tracking Adolescents’ Individual Lives [38]). (2) Children meet at least 2 of 3 criteria of our High Risk Index that was developed in the ARIADNE sample: (a) being female, (b) having two affected parents, (c) having a parent with (past) suicidal behaviour.

Exclusion criteria (offspring): mental retardation; not speaking Dutch fluently; severe alcohol or substance use disorder; current diagnosis of a mental disorder that warrants regular treatment. Children with a history of a mental disorder are included, as well as children with a current disorder that had sufficiently been treated (e.g. in the case of stable medication for ADHD).

### Procedure

**Figure 1:**
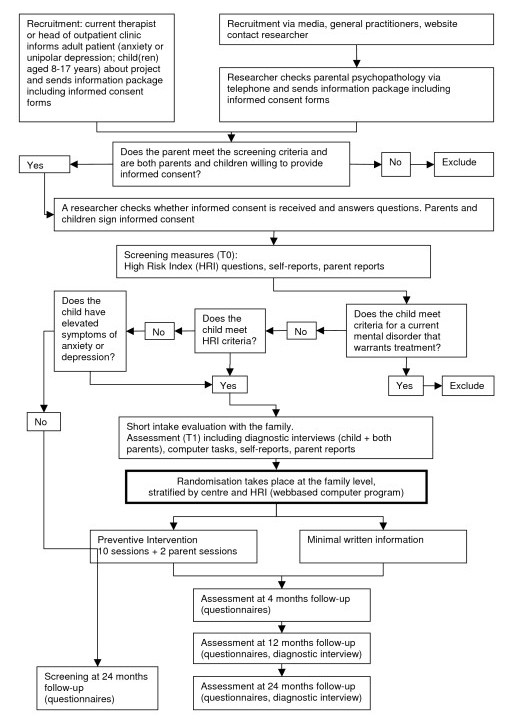
Flow diagram.

The study has two phases, namely the screening phase and the intervention phase. Details on the procedure are described in the flow diagram (Figure 1).

#### Referral and recruitment

Adult patients are recruited through mental health services, general practitioners, and by media (newsletters, social media, website, media attention through interviews for newspapers and radio). Adult and child mental health services participate in the recruitment of participants. In child mental health services, parents are included if they have an anxiety or mood disorder themselves. All children of these parents can participate in the study.

#### Informed consent

Adult patients are informed about the study in two ways. First, patients within a mental health care centre (adult or youth) are informed about the study by their therapist by a full information letter, containing all information for both the parents and the children. If the patient is interested in participating, he or she can contact the researcher. The researcher then checks whether the family has understood the information on the trial. If so, the consent form is signed by both parents and children, and the family is invited for the first screening. Separate informed consent forms are available for parents, adolescents (secondary school) and children (primary school). Even though informed consent is not warranted under the age of 12 in the Netherlands, we do have younger children sign informed consents so that we are sure they are fully informed about the study.

Second, patients may contact the researchers directly, after having received information about the study via their general practitioner, the media, or the website. In that case, the researcher directly informs the family members about the study by way of the information letters described above. We then check the status parent’s past and current psychopathology through a brief telephone interview. Note that for all parents the exact diagnosis is obtained during the assessments through a structured diagnostic interview (CIDI).

Participants can always draw back from participating in the study, at any time during the assessment or intervention, without giving a reason for their withdrawal. Withdrawal from the trial will not affect the regular mental health care for either themselves or their children.

#### Randomisation

Since outcome may be dependent on treatment centre and the pathway of inclusion (through elevated symptoms versus no symptoms/HRI only), we decided to work with stratified allocation to treatment condition to ensure a balanced number of participants over conditions. A web-based computer program allows for dynamic on-the-spot randomisation. Randomisation is based on the defined strata as well as on the assigned treatment condition of earlier subjects within the strata. To prevent conscious or unconscious influence on the recruiting team, we choose to conceal the exact details of the randomisation process at this stage (following the recommendations of the CONSORT guidelines [39]). Randomisation takes place after the whole assessment has been completed, so that the first assessment is not dependent on the participant’s knowledge of treatment condition. The research assistant assigns a unique code to each participant. In case one family has several high risk children, the youngest child is entered for randomisation, and the siblings are assigned to the same condition. The research assistant then enters the number of the participant in the web-based randomisation program, and at that moment the computer randomly assigns the treatment condition (active intervention or minimal information; 1:1).

#### Intervention

The intervention has 10 child sessions and 2 sessions for the parents. To strengthen the effectiveness of our intervention, it is based on a theoretical framework including both risk and protective factors (as recommended by Cuijpers [24]).The following themes are addressed: family functioning and social network, being proud of strengths, focus on positive emotions and events, problem solving, and breaking the cycle of avoidance behaviour. The latter will either be focussed on behavioural activation (indication for depressive symptoms [35]) or on exposure exercises (indication for anxiety symptoms). The therapist addresses each of the themes in the first sessions and then elaborates on the most appropriate module(s) for each child. In collaboration with the child and the parents, the child chooses 10 steps to work on throughout the sessions. These steps are in line with the aforementioned themes, and could encompass engaging in specific activities, exposure exercises, and exercises on strengths and resilience. Throughout the sessions, the child regularly monitors the frequency and type of activities (categorised as either alone or with others, and either at home or elsewhere). Ideally, children should have satisfying activities in each of these four categories. This registration is available for mediation analyses. The first session is with the parents only. The therapist makes a plan with the parents on how to give age-appropriate information on the parental anxiety or mood disorder to the child in the next session. Parents also receive information on the possible effect on offspring and protective factors against future child psychopathology (such as having a parent or key figure they can trust, and openness in the family). Two more parent sessions further elaborate on positive parenting and defining the social network in terms of social support for the family.

### Assessments

#### Assessment schedule

Baseline assessment takes place at T0 and comprises questionnaires on the child’s and the parent’s wellbeing (see Table 1). This first screening is used to define the high-risk study population. At T4, all children of parents that were willing to participate will be assessed again with the same measures. The latter is important to evaluate the validity of the initial selection of high risk individuals. If a relatively large proportion of the low-risk group nevertheless has developed a mood or anxiety disorder, this would imply that for future prevention programs the selection strategy should be reconsidered. Selection for the intervention phase is based on the HRI and the RCADS (see Figure 1).

**Table 1:** Assessment schedule for screening (T0) and 24 months follow-up screening

**Measures**	**Child**	**Parent 1**	**Parent 2**
Demographics (+ HRI)		X	
Child anxiety and depression (RCADS)	X	X	
Child impairment (BIS)		X	
Child externalizing symptoms (SNAP)		X	
Parent positive and negative affect (PANAS)		X	X
Optimism (LOT/YLOT)	X	X	X

If a child qualifies for “ultra high risk” based on the screening, he or she may enter the intervention phase of the study. The pre-intervention assessment encompasses structured diagnostic interviews with the child and both parents, as well as a number of relevant other measures (see Table 2). Assessments in the intervention phase are planned for month 0 (T1, at the start of the intervention), month 4 (T2), month 12 (T3), and month 24 (T4). A subset of the instruments is also scheduled for month 6 and 18.

**Table 2:** Assessment schedule for intervention phase

**Measures**	**T1**	**T2**	**T3**	**T4**	**Child**	**Parent1**	**Parent2**
Child diagnoses (DISC)	X	-	X	X	X	X	-
Child anxiety and depression (RCADS)	X	X	X	X	X	X	-
Child impairment (BIS)	X	X	X	X	X	-	-
Child externalizing symptoms (SNAP)	X	X	X	X	X	-	-
Child attachment (IPPA)	X	X	X	X	X	-	-
Implicit child attachment (ABT)	X	-	-	-	-	-	-
Child coping (CSLK)	X	X	X	X	X	-	-
Child attributions (CASQ)	X	X	X	X	X	-	-
Child optimism (YLOT)	X	X	X	X	X	-	-
Child self-esteem (RSES)	X	X	X	X	X	-	-
Expressed emotion (FMSS)	X	-	-	-	-	X	X
Implicit Self-associations (EAST)	X	-	-	-	-	-	-
Child executive functions (BRIEF)	X	-	-	-	-	X	-
Child temperament (EATQ)	X	X	X	X	-	X	-
Parent positive and negative affect (PANAS)	X	X	X	X	-	X	X
Parent depression (BDI)	X	X	X	X	-	X	X
Parent anxiety (BAI)	X	X	X	X	-	X	X
Parent substance misuse (AUDIT)	X	X	X	X	-	X	X
Parent optimism (LOT)	X	X	X	X	-	X	X
Parent psychopathology (CIDI)	X	-	-	-	-	X	X
Cost Effectiveness*	X	X	X	X	-	X	X
Quality of life (EQ-5D)*	X	X	X	X	X	X	X

* Cost effectiveness measures and EQ-5D will also be administered at 6 and 18 months.

Note that T1 = pre-intervention, T2 = 4 months follow-up, T3 = 12 months follow-up, and T4 = 24 months follow-up.

#### Measures

##### Primary outcome

Our primary outcome is the time to onset of depression or anxiety disorders in the offspring, based on the Child version of **Diagnostic Interview Schedule for Children** Version IV NIMH DISC-IV; [40], which is a highly structured diagnostic assessment instrument designed to gather symptom presence for child and adolescent psychiatric disorders based on the symptoms and criteria variables as defined in the Diagnostic and Statistical Manual of Mental Disorders DSM-IV; [41]. The computerized DISC is administered via a computer (the interviewer reads questions from the computer screen and enters responses directly into the computer) and scored by computer algorithm [42]. There are parallel versions of the instrument: the DISC-P for parents, and the DISC-C for direct administration to children. In the present study the DISC-C is used to assess anxiety disorders and depression, and a brief assessment of alcohol and drug use. The DISC-P is used as a parent report of children’s anxiety and depression symptoms as well as questions about whole life symptomatology. At the 12- and 24-follow-up assessments, the interviewer addresses the past 12 months. If any disorder was present, the interviewer makes an estimation of the time of onset and the duration of the disorder. The interviewer combines information retrieved from the DISC with the **Weekly Emotions Diary for Youth (WEDY;** Festen & Nauta, Weekly Emotions Diary, unpublished manuscript**)** as input. All children included in the intervention trial are asked to keep a weekly diary during the study, giving scores of three items: anxiety, sad mood, and happiness on a 0–5 scale. This chart was developed for the purpose of the current study, primarily as a helpful tool to obtain a good estimate of the onset and duration of a disorder (if present by DISC-criteria), since children may not always have an adequate sense of time when retrospectively reporting over the past year. Children are encouraged to fill out the diaries either on paper or through the internet in a web-based diary. They receive an incentive of 1 euro per month for completing the weekly diary, with a maximum of 25 euros.

##### Secondary outcomes

As secondary outcomes to our study, we will investigate the number of days with a disorder (based on the DISC-IV), child and parent anxiety and depressive symptoms, quality of life, and cost-effectiveness.

The **Revised Child Anxiety and Depression Scale** (RCADS; RCADS; [43]) is a 47-item self-report and a parent-report questionnaire, with scales corresponding to separation anxiety disorder, social phobia, generalized anxiety disorder, panic disorder, obsessive-compulsive disorder and major depressive disorder.

The **Inventory of Depressive Symptomatology** IDS; [44] is a 30-item measure of depressive signs and symptoms in adults. In the current study the Dutch translation of the self-report (SR) version was used to assess current depressive symptoms of the parents

The **Beck Anxiety Inventory** BAI; [45] is a 21-item self-report instrument that assesses the overall severity of anxiety in adults. In this study the BAI is used to assess current anxiety symptoms of parents.

To assess quality of life, we included the EuroQol (**EQ-5D**[46]), being a brief, easy to administer questionnaire comprised of 2 components: a description of the respondent’s own health using a health status classification system with 5 items and a rating of “own health” by means of a visual analogue scale (VAS; 0–100). We included a child version, a parent version and a regular adult self-report in the current study.

Information on healthcare consumption will be registered with a comprehensive questionnaire capturing (mental) healthcare consumption and other illness-related economic consequences for society. The instrument is administered to the parents and assesses various cost aspects of the child and each parent, including contacts with healthcare professionals, informal care and absence from school or work. The instrument is a revision and adjustment of the youth care version of the TIC-p [47] and has been adopted to the specific situation of the current study. The intervention costs are also monitored including therapist time spent on the actual intervention, the preparation of sessions, course material, travelling costs, and the costs of the training of the therapists.

##### Mediators and moderators of change

To examine potential mediators and moderators of change we assessed the following measures focused on child characteristics and parent characteristics:

Measures on the child’s characteristics

The **Youth Life Orientation (YLOT;**[48]**)** is a 12-item measure of dispositional optimism and pessimism that was developed as a child analogue of the widely used Life Orientation Test.

The **Children’s Attributional Style Questionnaire - Revised** CASQ-R; [49] is a 24-item shortened measure designed to assess children’s causal explanations for positive and negative events.

The **Rosenberg Self-Esteem Scale** RSES; [50] is developed as a 15-item self-esteem scale for children and adults and translated into different languages.

An Extrinsic Affective Simon Task **(EAS**T; [51]) is a computerized reaction time task to indirectly measure attitudes. The current EAST was designed for this study and intends to assess the strength of the children’s automatic associations between themselves and sad (or happy) mood, as well as anxious (or calm) feelings. It is a categorization task, during which target words like “myself”, “them”, “anxious”, or “table” appear in the middle of the computer screen, and children have to assign the target words to one of the target labels (me, not-me, feeling, or object) that are set at either the left or the right side of the screen. In correspondence, children press a right or left response key as quickly as possible. The underlying principle is that a person will be faster in categorizing anxiety or depression words to the key that is defined as both “feeling” and “me” label if the person regards him or herself as anxious or depressed. In contrast, the person may be slower in categorizing the words related to calmth or happiness to a key that is defined as both a “feeling” and “me” label. These types of automatic anxious- and depressed self-associations have been found to be predictive of symptomatology in adults [37].

The **Coping Strategies Checklist for Children** CCSC-R1; Dutch version: CSLK; [52,53] contains 14 subscales, including a variety of cognitive coping strategies. Earlier research in offspring has shown that it may be important to distinguish between coping at home and coping elsewhere [28]. Therefore, we administered two versions of the coping questionnaire. The scale contains five coping dimensions (Seeking understanding, Control, Optimism, Wishful thinking, Support for feeling and Support for actions).

The **Brief Impairment Scale** BIS; [54] is a multidimensional scale of functional impairment for children and adolescents. The BIS is a 23-item instrument that evaluates three domains of functioning: interpersonal relations, school/work functioning, and self-care/self-fulfillment.

The **Behaviour Rating of Executive Functioning** BRIEF; [55] is a 86-item standardized rating scale used to assess children’s executive functions in home and school environments. The **Early Adolescent Temperament Questionnaire – Revised version** (EATQ-R; [56]) is a parent-report questionnaire that was developed to measure child temperamental aspects associated with self-regulation. Three factors were included in the present study with a total of 44 items, namely Effortful control (including activation control, attentional control, and inhibitory control), Negative affect (including Fear, Frustration, and Shyness) and High intensity pleasure/Surgency.

The **SNAP-IV Parent Rating Scale** (SNAP; [57]) is a parent report questionnaire including 18 items for ADHD (inattention and hyperactivity/impulsivity) and 8 items for ODD symptoms.

The Dutch version of the **Inventory of Parent and Peer Attachment** IPPA; [58,59] is an 11-item self-report instrument that is used to assess attachment to each parent and one significant other.

Mother-child attachment was also administered through a computer task, the **Attentional Breadth Task**[60], that was programmed in e-prime. The task is based on the assumption that information processing may be distorted in individuals with insecure attachment when it comes to attachment-relevant information. Insecurely attached children may be more alert to stimuli that are related to the mother, and may be more likely to have a smaller attention span when the mother is involved. During the task, children are shown a picture of either their mother or an unfamiliar woman, as well as a dark dot that appears either close to the picture or further away. Children have to report on whether the woman was their mother or the unfamiliar woman, and must then identify where the dot appeared. For further details and specifications, see [60]. Attentional Narrowing Indices (ANI) are presumed to be a proxy for implicit attachment security and can be derived from the child’s reaction times (ANI = stimulus close to picture – stimulus far from picture).

The **Five Minute Speech Sample**[61] is used to assess perceived expressed emotion by the father and the mother. Each parent is asked to talk for five minutes about his or her child and the relationship they have with their child. After coding the text, two components can be derived: criticism (CRIT) and emotional overinvolvement (EOI).

##### Measures on parental well-being and psychopathology

The WMH Survey version of the World Health Organization (WHO) **Composite International Diagnostic Interview** WMH-CIDI, now CIDI 3.0; [62] is a comprehensive, fully-structured interview designed to be used by trained lay interviewers for the assessment of mental disorders according to the definitions and criteria of DSM-IV [41]. We used the Dutch translation [63] in a computerized version, containing of the following sections: Depression, Mania, Panic Disorder, Social Phobia, Separation Anxiety Disorder, Specific Phobia, Generalized Anxiety Disorder, Obsessive Compulsive Disorder.

The **Alcohol Use Disorders Identification Test**[64,65] was developed by the World Health Organization (WHO) as a simple method of screening for excessive drinking. Seven of its items reflect harmful and dependent drinking, while three items assess alcohol consumption behaviour in terms of quantity and frequency of drinking.

The **Positive and Negative Affect Schedule** (PANAS) is a 20-item self-report measure of positive and negative affect [66]. Negative Affect and Positive Affect reflect dispositional dimensions, with high NA epitomized by subjective distress and unpleasurable engagement, and low NA by the absence of these feelings. By contrast, PA represents the extent to which an adults experiences pleasurable engagement with the environment.

### Sample size calculation

Our sample size calculation is based on the conventional significance (alpha) and power (1-beta) levels of 0.05 and 0.80 respectively, planning one-sided testing. We assume a baseline incidence rate of mood and anxiety disorders of 0.11/person*year for the control condition, based on our own unpublished analyses in high risk offspring from the ARIADNE study; in the ultra-high risk subgroup of children with 2 or 3 risk factors we found a 10-year cumulative incidence of anxiety- and mood-disorders of 0.67 (time frame: age 10–20), which corresponds to an incidence rate of 0.11/person*year, assuming a constant rate. With an intended follow-up duration of 2 years, we would then need a minimum of 81 participants per condition to give our study sufficient power to detect a clinically meaningful effect of the intervention on the time to onset of episodes of mood- and anxiety disorders. We compared our estimated incidence rates with two prevention studies. Clarke et al. [13], who studied the effect of a preventive program in subsyndromal offspring of depressed patients, found a 1 year cumulative incidence of 0.25 for depression alone (rate 0.29/person*year). Therefore, our incidence assumption is relatively conservative. We assume a risk reduction of 70% for the treatment condition, based on the Clarke study; the 1 year cumulative incidence of depression in the intervention group is 0.08, compared to 0.25 in the control group (unadjusted hazard ratio 0.29). The reported adjusted hazard ratio in this study is 0.18. Dadds et al. [67] report a hazard ratio of 0.22 in favor of a program aimed at preventing anxiety disorders in children. Again, we choose the more conservative estimate. Nevertheless, realizing that above-mentioned assumptions remain uncertain and that participants may drop out, we decided to include another 25%, resulting in 2 groups of 102 children for the intervention phase (N = 204).

Including 554 children for the screening (T0), we estimate that 388 children will be in the ultra high risk group (70%) and 166 children in the no ultra high risk group (30%). We anticipate 20% of the eligible children to already suffer from a mental disorder that warrants treatment, and another 20% to not want to participate in the intervention phase of the study for various reasons (leaving N = 204 for randomization). In order to include 554 children in the screening, we anticipate that we need to select 2770 adult patient files (80% no children in the right age range or no interest in participating in the study). This percentage was based on our earlier experience with the epidemiological ARIADNE study, following offspring over time.

### Statistical analyses

Survival analysis will be used to answer the first research question (effect of treatment on the possible onset of depression or anxiety disorder). It encompasses a wide variety of methods for analyzing the timing of events. The logrank test (sometimes called the Mantel-Cox test) is a hypothesis test and will be used to compare the ‘survival’ distribution between the intervention and the control group. The measurement is the time to event (clinical onset of anxiety disorder or depression). Therefore ‘survival’ is ‘no onset of an anxiety or mood disorder’ in the study period of 2 years.

All data will be analyzed using the intent-to-treat principle. Multilevel analysis is a general term referring to statistical methods appropriate for the analysis of data sets comprising several types of units of analysis. Multilevel analysis holds two important features that are relevant to our data, namely the handling of missing data, as well as the possibility to work with dependent data. In our case, siblings are dependent (“nested”) data and can be analyzed as such. Intention to treat analyses are done to avoid the effects of crossover and drop-out, which may break the randomization to the treatment groups in the study. Mediators and moderators can be included as explanatory variables into the multilevel model.

#### Economic evaluation

An economic evaluation will be conducted alongside the current study to assist future policy making and resource allocation. The economic evaluation focuses on the balance between costs and health outcomes of the treatment conditions. Both short-term and long-term consequences are taken into account.

For the short-term economic analyses, data will be collected prospectively during the 24 months of this study. Both a cost-effectiveness and cost-utility analysis will be conducted to provide information on the short-term economic outcomes. In the cost-effectiveness analysis, the incremental costs per depression & anxiety-free year gained are assessed. The cost-utility analysis will provide information on the incremental costs per Quality Adjusted Life Year (QALY) gained. QALYs will be derived from the EQ-5D [46], which is a brief instrument commonly used in economic evaluations. Since the economic evaluation is conducted from a societal perspective, both medical costs and costs outside the healthcare sector will be assessed. Costs and health outcomes will be discounted in accordance with Dutch guidelines. Bootstrap analyses are planned to provide information on the uncertainty surrounding the economic results. Subsequently, cost-effectiveness acceptability curves will be used to inform decision-makers on the probability that the intervention is cost-effective.

We will also conduct a long-term analysis. The relatively short follow-up period of the prospective part of the study has the drawback of potential underestimation of future beneficial effects of the program. In the proposed long-term analysis we will use decision analytic modeling to explore the cost-effectiveness of the program with a longer time horizon (10 yrs). Primary data from the trial will be combined with secondary data sources, parameter-estimates from the literature, and where necessary other sources such as expert opinions. Where needed, meta-analyses will be performed to aggregate available literature data. A Markov-type decision model will be used in the analysis, which is in general well suited to model chronic diseases, characterized by repeated relapses and remissions over time. In a Markov- (or state-transition) model, health states are defined, together with the probabilities of making the transition from one health state to another. These models simulate the accumulation of health/quality of life effects and costs over time under different strategies e.g. [68]. We plan to use data from NESDA (the Netherlands Epidemiological Study on Depression and Anxiety [69]), a large ongoing naturalistic study in patients with mood and anxiety disorders, to model long-term disease history (e.g. to model duration and frequency of episodes or transitions between mood- and anxiety disorders). The decision analysis will also look at long-term productivity effects of the program; Fergusson et al. [70] demonstrated, for example, a dose‐response relationship between number of depressive episodes during adolescence and productivity later in life. Modeling allows for exploration of cost-effectiveness of the program under study in different scenarios, e.g. to model cost-effectiveness under different assumptions regarding the duration of the treatment effect. Sensitivity analyses can also be used to reveal the parameters that are most crucial to the outcome of the study, which will help to efficiently allocate future research resources.

### Ethical approval

The Medical Ethical Committee of the University Medical Center in Groningen gave ethical approval for the study (nr 2009.200). Multi-center ethical approval was obtained for the sites in Groningen (UMCG/Accare), Friesland (GGZ-Friesland), and Leiden (LUMC/Curium-LUMC).

## Discussion

Given the high prevalence of anxiety and mood disorders in offspring of adults with these disorders, prevention interventions are needed to prevent internalising mental disorders in children and adolescents. The current STERK-study is a randomised controlled prevention trial in high-risk offspring (aged 8–17 years) of anxious and depressed patients. We select youth on either elevated symptoms or on the basis of the High Risk Index that we developed in an earlier epidemiological offspring study (ARIADNE). The present study is the first to focus on both anxiety and depression, since these disorders are often comorbid, and since epidemiological findings in offspring show that anxious and depressive symptoms are prevalent in offspring of patients with anxiety disorders [30] and just as well in patients suffering from unipolar depression [31]. With this study, we hope to contribute to the prevention of mental disorders in offspring, as well as to the knowledge on mediators and moderators of change.

Developing prevention studies, getting funding, gaining ethical approval, and including participants is a time-consuming trajectory. For the current study/project, the grant application was in 2007, the grant was awarded in 2008, and we received ethical approval by the end of 2009. The inclusion of participants into the project is still ongoing. Due to this timeframe, we have not been able to incorporate some of the most recent findings into our design. In fact, the current design is an adaptation of an earlier design. In the first wave of the study, we only included adult patients that were currently in treatment, and we excluded all children with a history of mental illness. However, we noticed that quite some eligible and interested participants were excluded in this way. In addition, the Garber study [14] got published in 2009, showing that prevention interventions may be only fruitful if parents were remitted in terms of their own mental health problems. Therefore, we decided to broaden the inclusion scope of our trial (approved of by both the governmental funding organization ZonMw and the Medical Ethical Committee).

A preventive intervention of this kind should not be a stand-alone in clinical practice. Many youth departments primarily work on the youth’s psychopathology and hardly identify parental psychopathology. Likewise, mental health centers for adult patients often do not consider the mental health status of offspring. During the process of implementing this study, we noticed that therapists are often unaware if the adult patient has children. The electronic patient files usually do not map age or emotional well being of children. It would be helpful in clinical practice to pay attention to the psychopathology in the family as a whole, and to have more cooperation between the youth and adult departments for cross-referrals. In line with Garber’s findings it might be helpful to address the emotional well being of the children in the remitted instead of the acute phase of the parent’s disorder. The STERK-study may provide valuable tools within such an infrastructure: a way of screening for high-risk children, as well as an intervention aiming at symptom reduction and resilience.

## Competing interests

During the years 2007–2012 **W.A. Nolen** has received grants from the Netherlands Organisation for Health Research and Development, the European Union, the Stanley Medical Research Institute, Astra Zeneca, Eli Lilly, GlaxoSmithKline and Wyeth; has received honoraria/speaker’s fees from Astra Zeneca, Lundbeck, Pfizer and Wyeth; and has served in advisory boards for Astra Zeneca. All other authors report no competing interests.

## Authors’ contributions

MN, HF, SdV, and CR drafted this paper which was added to and modified by all other authors. MN and HF developed the content of the STERK-intervention in collaboration with therapists at Accare. All authors contributed to the design of the study and MN, DS and SdV to the analytic strategy. All authors read and approved the final manuscript.

## Pre-publication history

The pre-publication history for this paper can be accessed here:

http://www.biomedcentral.com/1471-244X/12/31/prepub
